# TGFBR-IDH1-Cav1 axis promotes TGF-β signalling in cancer-associated fibroblast

**DOI:** 10.18632/oncotarget.20861

**Published:** 2017-09-13

**Authors:** Xiaodan Hou, Jieying Zhang, Yongbin Wang, Wujun Xiong, Jun Mi

**Affiliations:** ^1^ Department of Biochemistry and Molecular Cell Biology, Shanghai Key Laboratory of Tumor Microenvironment and Inflammation, Shanghai Jiao Tong University School of Medicine, Shanghai, China; ^2^ Shanghai East Hospital, Affiliated to Tongji University, Shanghai, China; ^3^ Center of Systems Medicine, Chinese Academy of Medical Sciences, Suzhou Institute of Systems Medicine, Suzhou, China

**Keywords:** IDH1, TGFBR, TGF-β signalling, Cav1, alpha-ketoglutarate

## Abstract

TGF-β signalling plays an important role in fibroblasts activation and tumour progression. Here, we report that the TGFBR-IDH1-Cav1 axis promotes TGF- β signalling in fibroblasts. Our data demonstrated that IDH1 was downregulated by TGF-β signalling in fibroblasts, and downregulation of IDH1 increased cellular concentration of α-ketoglutarate (α-KG) by accelerating glutamine metabolization. Interestingly, α-KG suppressed Cav1 expression through reducing the trimethylation of histone H3K4. Furthermore, Cav1 downregulation inhibited TGFBR protein degradation. In turn, the activated TGFBR promoted TGF-β signalling. These findings demonstrated that metabolic enzyme IDH1 regulates TGF-β signalling by feedback mechanism through α-KG and TGFBR-IDH1-Cav1 axis is important for TGF-β signalling.

## INTRODUCTION

The tissue and cellular homeostasis are controlled by different signalling pathway, which is a balanced crosstalk in intracellular signal transduction. Understanding the timely and special signal pathway involved in the physiological and pathological process will develop new strategies for clinical therapeutics such as cancer treatment. Transforming growth factor β (TGF-β) signalling participates in diverse cellular processes such as proliferation, differentiation, apoptosis and extracellular matrix formation, depending on the cell type. In wound or tumour tissue, TGF-β signalling also plays an important role in fibroblasts activation. TGF-β initiates these diverse cell type-dependent responses through forming a heterodimer with the type I/II TGF-β receptor (TGFBR). Once TGF-β binds to TGFBRII and forms the TGFBRII/I heterodimer, TGFBRI is activated and phosphorylates the conserved motif (Ser-Ser-X-Ser) of Smad2 and Smad3 at their extreme C-terminal ends; phosphorylated Smad2 and Smad3 form a complex with Smad4 [1]. The activated Smads complex then translocates into the nucleus and, in conjunction with other nuclear cofactors, regulates the transcription of a large number of target genes [2, 3]. This pathway is known as canonical TGF-β signalling. Distinct from canonical TGF-β signalling, TGF-β-activated TGFBR also phosphorylates p38 and Erk1/2, which are belonged to non-canonical TGF-β signalling. The interference with molecule(s) on the TGF-β pathway, including ligand-receptor binding and the degradation of TGFBR, regulates the strength of TGF-β signalling. Cav1, a protein which is a main component of the caveolae plasma membranes, was previously reported to mediate the degradation of TGFBR[4, 5].

Recent reports have shown that TGF-β increased Glut1 expression and glucose uptake in the EMT transformed breast cancer MCF-7 cells [6]; and Hexokinase 2 (HK2) was also increased in TGF-β-activated fibroblasts [7]. All these observations suggest that TGF-β signalling participates in metabolic regulation. However, it's not clear whether metabolic alteration affects TGF-β signalling.

Isocitrate dehydrogenase 1 (IDH1), a member of the isocitrate dehydrogenase family, is mostly located in the cytoplasm and converts isocitrate to α-ketoglutarate (α-KG) in a NADP^+^-dependent manner. α-KG is an important allosteric regulator of dioxygenases, which involve in diverse cellular processes, such as the synthesis of antibiotics and collagen [8], the control of oxygen homeostasis [9, 10], DNA modification and histone demethylation [11]. Moreover, more than half of dioxygenase family members regulate gene expression [12–14].

In some glioblastoma and acute myeloid leukemia patients, the codon 132 mutation in the active site of IDH1 was detected. The mutation results in a loss of normal enzymatic function and turns into a new enzymatic activity that transforms α-KG into 2-hydroxyglutarate (2-HG). The oncometabolite 2-HG accumulates in the cell and acts as a competitive inhibitor of α-KG in many cellular reactions. Our data showed that IDH1 could regulate TGF-β signalling through α-KG-dependent dioxygenase.

## RESULTS

### TGF-β signalling downregulates the expression of IDH1

**Figure 1:**
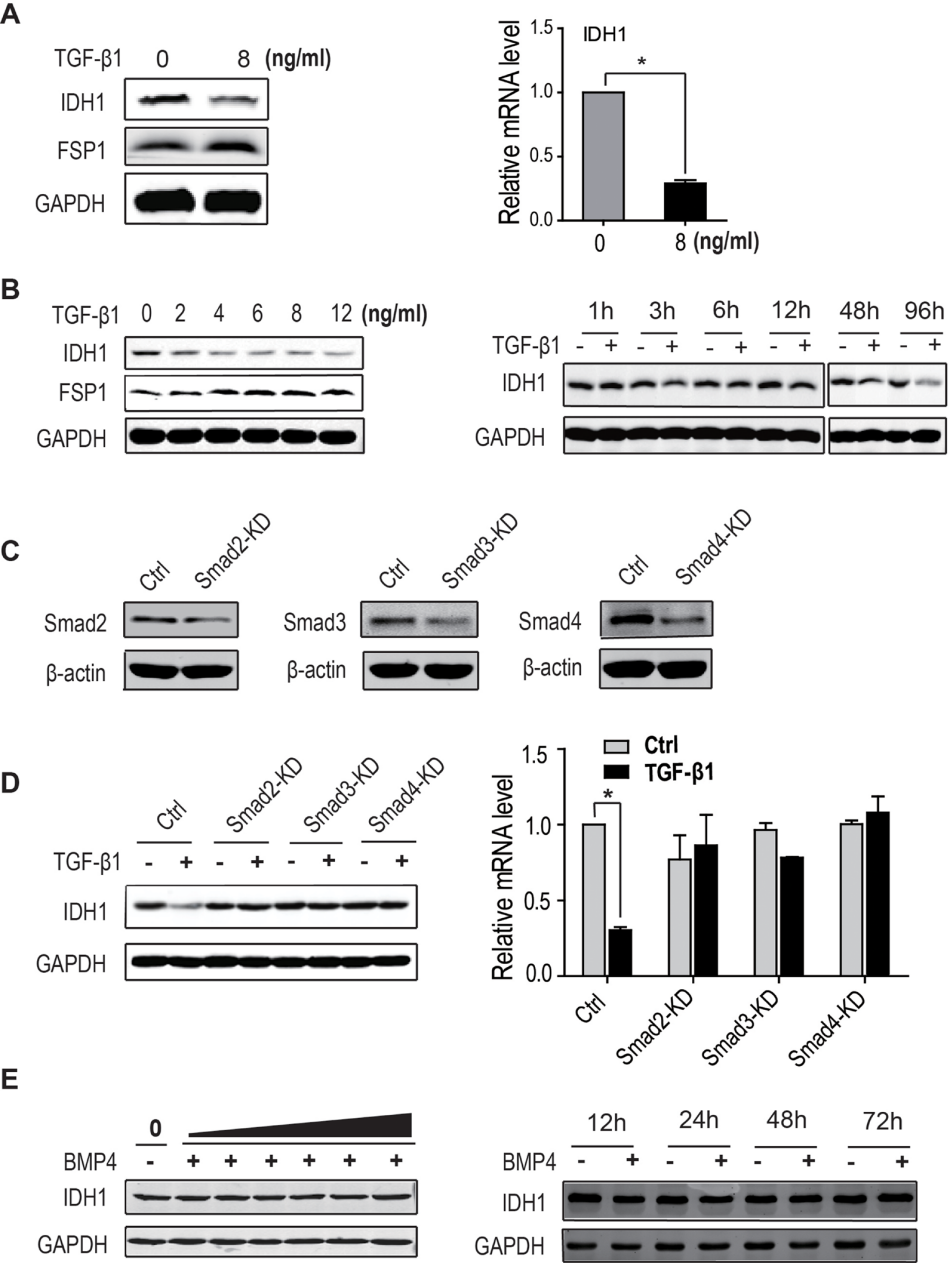
IDH1 is downregulated by TGF-β signalling (**A**) The protein (Left panel) and mRNA levels of IDH1 (Right panel) decreased in TGF-β-treated primary fibroblasts. Primary fibroblasts were treated twice with 8 ng/ml of TGF-β1 for 48 hours, and mRNA levels were analysed by quantitative PCR and normalised to GAPDH (*n* = 3, *p < 0.01). (**B**) The protein levels of IDH1 gradually decreased with increasing TGF-β1 concentration. Primary fibroblasts were treated twice with TGF-β1 at the indicated concentrations (0, 2, 4, 6, 8, and 12 ng/ml) for 48 hours (left panel). Meanwhile, the IDH1 protein level was analysed in fibroblasts treated with 8 ng/ml TGF-β1 for the indicated times (0, 1, 3, 6, 12, 48, and 96 hours) (Right panel). (**C**) Smad2, Smad3 or Smad4 were silenced respectively in primary fibroblasts. (**D**) The protein and mRNA levels of IDH1 significantly decreased in primary fibroblasts treated with TGF-β1, but not in fibroblasts depleted of Smad2, Smad3 or Smad4 in the presence or absence of TGF-β1. The mRNA levels were normalised to GAPDH (*n* = 3, *p < 0.01). (**E**) The protein levels of IDH1 were not affected by BMP4 treatment. Primary fibroblasts were treated twice with BMP4 at the indicated concentrations (0, 2, 4, 6, 8, 10, and12 ng/ml) for 48 hours (Left panel). Meanwhile, the IDH1 protein levels were also analysed in fibroblasts treated with 12 ng/ml of BMP4 for the indicated times (0, 12, 24, 48, and 72 hours); fibroblasts without BMP4 treatment were considered control (Right panel). All Western blots were independently repeated three times, and RT-PCR data are presented as the means ± SD.

Our previous deep sequencing data showed that the expression of IDH1 was decreased in TGF-β1-activated fibroblasts, which was verified by the expression of FSP1, a specific marker of activated fibroblasts (Figure 1A). However, the further study showed that IDH1 did not decrease in PDGF-activated fibroblasts (Supplementary Figure 1). These data indicate that the expression of IDH1 is only regulated by TGF-β1 signalling. To further confirm this TGF-β-induced downregulation of IDH1, dose-response and time-course experiments were performed. Our data showed that the protein level of IDH1 gradually decreased with increasing TGF-β1 concentration (0, 2, 4, 6, 8, and 12 ng/ml) 96 hours after treatment. In the time-course experiment, the expression of IDH1 was detected in fibroblasts treated with 8 ng/ml of TGF-β1, and the protein level of IDH1 began to decrease 12 hours after treatment and reached its lowest level at 96 hours (Figure 1B). Moreover, our data showed that this decrease in IDH1 protein level did not result from enhanced protein degradation (data not shown). These data demonstrated that IDH1 was downregulated in TGF-β1-treated fibroblasts.

To determine whether this downregulation of IDH1 is dependent on canonical TGF-β signalling, fibroblasts depleted of Smad2, Smad3 or Smad4 were treated with or without TGF-β1. Our data showed that the protein level of IDH1 significantly decreased in control fibroblasts treated with TGF-β1, but not in fibroblasts depleted of Smad2, Smad3 or Smad4, with or without TGF-β1. Quantitative PCR also showed that the mRNA level of IDH1 was only reduced in TGF-β1-treatedcontrol fibroblasts. These data suggest that TGF-β1-induced suppression of IDH1 expression is dependent on the canonical Smad pathway (Figure 1C and 1D).

To exclude the possibility of Smad1/Smad5 signalling participating in the regulation of IDH1, the protein levels of IDH1 were analysed in fibroblasts treated with BMP4, which specifically activates Smad1/Smad5 signalling. Our data showed that the protein levels of IDH1 did not change with increasing BMP4 concentration (0, 2, 4, 6, 8, 10, and 12 ng/ml); moreover, no significant difference was observed in IDH1 expression at the indicated times (12, 24, 48, and 96 hours) after BMP4 treatment (Figure 1E). In brief, these data demonstrated that downregulation of IDH1 was regulated by canonical TGF-β signalling.

### Downregulation of IDH1 enhances TGF-β signalling

**Figure 2:**
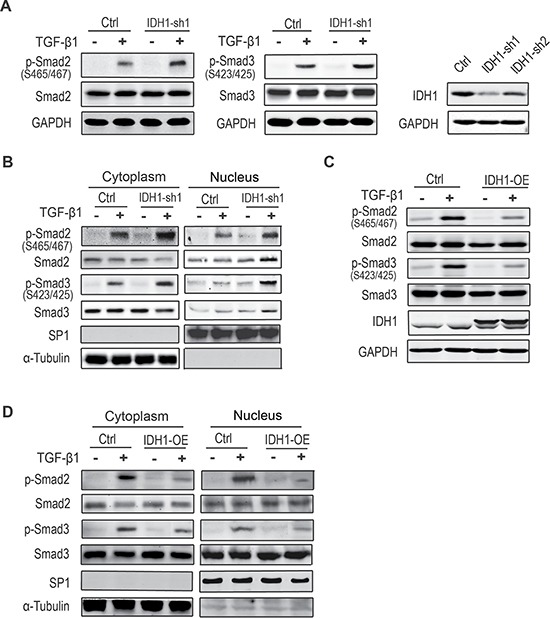
Downregulation of IDH1 enhances canonical TGF-β signalling (**A**) The C-terminal phosphorylation levels of Smad2 and Smad3 were enhanced in fibroblasts depleted of IDH1 after TGF-β1 treatment. All fibroblasts treated with or without TGF-β1 (8 ng/ml for 1 hour) were analysed by Western blot. The short hairpin RNA knockdown efficiency of IDH1 was analysed by Western blotting (right panel). (**B**) The phosphorylation levels of Smad2 and Smad3 increased in both fractions of cytoplasm and nuclei in fibroblasts depleted of IDH1, which were treated with or without TGF-β1 (8 ng/ml) for 1 hour before analysis. SP1 and α-tubulin were considered cytoplasm marker and nucleus marker, respectively. (**C**) The phosphorylation levels of Smad2 and Smad3 were decreased in IDH1-overexpressing fibroblasts, which were treated with or without TGF-β1 (8 ng/ml) for 1 hour before analysis. (**D**) The phosphorylation levels of Smad2 and Smad3 were decreased in both fraction of cytoplasm and nuclei in fibroblasts overexpressing IDH1.

To determine the effect of IDH1 downregulation on TGF-β signalling, the total protein and phosphorylation levels of Smad2 and Smad3 were analysed in fibroblasts depleted of IDH1. After comparison of the knockdown efficiency of IDH1, fibroblasts infected with the shRNA1 were analysed. Our data showed that the C-terminal phosphorylation levels of Smad2 and Smad3 were enhanced in fibroblasts depleted of IDH1 after TGF-β1 treatment. However, IDH1 knockdown had little effect on the phosphorylation of Smad2 or Smad3 without TGF-β1 stimulation. Moreover, the total protein levels of Smad2 and Smad3 did not significantly increase in IDH1-knockdown fibroblasts compared to control fibroblasts (Figure 2A).

To further determine whether IDH1 affected TGF-β signalling, fractionation experiments were performed to determine if the phosphorylation levels of Smad2 and Smad3 were increased in the nucleus. The data showed that the phosphorylation levels of Smad2 and Smad3 were increased in both the cytoplasm and nuclei of fibroblasts depleted of IDH1 after TGF-β1 stimulation (Figure 2B). These observations suggest that IDH1 regulates the activation of canonical Smad pathway in response to TGF-β stimulation.

Meanwhile, the phosphorylation of Smad2/3 was also analysed in IDH1-overexpressing fibroblasts. Our data showed that the TGF-β-triggered-phosphorylations of Smad2 and Smad3 were suppressed in fibroblasts overexpressing IDH1, and there were no significant changes in the total protein level of Smad2 and Smad3 (Figure 2C). Moreover, the phosphorylation levels of Smad2 and Smad3 were decreased in both the cytoplasm and nuclei (Figure 2D).

All of these observations suggest that IDH1 regulates the phosphorylations of Smad2 and Smad3 and that the downregulation of IDH1 enhances TGF-β-activated canonical Smad signalling.

### IDH1 suppresses TGFBR degradation

**Figure 3:**
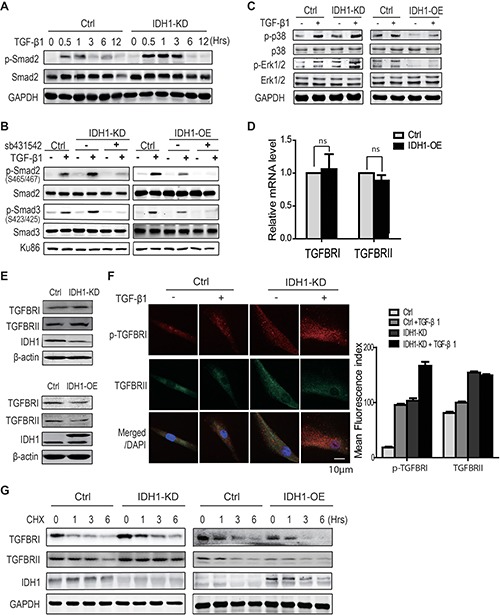
IDH1 suppresses TGFBR degradation (**A**) Western blot showing that IDH1 knockdown enhanced, but did not prolong, the TGF-β-induced phosphorylation of Smad2. Primary and IDH1 knockdown fibroblasts were treated with TGF-β1 (8 ng/ml) for the indicated times (0, 0.5, 1, 3, 6, and 12 hours). (**B**) Western blot showing the phosphorylation of Smad2 and Smad3 in IDH1-overexpressing and -knockdown fibroblasts with or without TGFBR 1 inhibitor (sb431542). Primary fibroblasts were treated with 8ng/ml of TGF-β1 for 1 hour after incubation with 10 μM sb431542 for 30 minutes. Ku86 was considered a loading control. (**C**) Western blot showing the phosphor- and total protein levels of p38 and ERK in IDH1-overexpressing or -knockdown fibroblasts treated with or without TGF-β1 (8 ng/ml) for 30 minutes. (**D**) RT-PCR showed the mRNA levels of TGFBR 1 and TGFBR II in IDH1 overexpression and control fibroblasts (*n* = 3; ns, *p* > 0.05). (**E**) Western blot showed the protein levels of TGFBR 1 and TGFBR II in IDH1 overexpression or silencing fibroblasts. (**F**) The immunofluorescence staining of TGFBR1 and TGFBR II. Fibroblasts were fixed and permeabilized; pictures are representative confocal microscopy images (40 ×). The mean fluorescence index was the average intensity analyzed in 100 cells using Image J (*p < 0.01, *n* = 100). (**G**) Western blot showed the protein level of TGFBR1 in IDH1 overexpression or silencing fibroblasts, which were treated with or without cycloheximide for the indicated times (0, 1, 3, 6 hrs).

To explore the mechanism by which IDH1 regulates the phosphorylations of Smad2 or Smad3, the phosphorylation level of Smad2 was analysed at the indicated time points (0, 0.5, 1, 3, 6, and 12 hours) after TGF-β1 treatment in fibroblasts with or without IDH1 knockdown. Western blot analysis showed that IDH1 knockdown enhanced the TGF-β-induced phosphorylation of Smad2 from 30 minutes to 3 hours (Figure 3A), suggesting IDH1 regulates the activity of the upstream kinase TGFBR.

To determine whether IDH1 regulates Smad signalling through TGFBR, fibroblasts depleted of IDH1 or overexpressing IDH1 were treated with the TGFBR 1 kinase inhibitor (sb431542). Our data showed that the inhibition of TGFBR1 abolished the TGF-β1-induced phosphorylations of Smad2 and Smad3 in both control fibroblasts and fibroblasts depleted of IDH1 (Figure 3B). Meanwhile, sb431542 also abolished the TGF-β1-induced phosphorylations of Smad2 and Smad3 in fibroblasts overexpressing IDH1 (Figure 3B). These data suggest that IDH1 regulates Smad activation through TGFBR.

Moreover, the phosphorylation levels of p38 and Erk1/2, two non-canonical targets of TGFBR, were also examined in fibroblasts overexpressing or depleted of IDH1. The effects of IDH1 on the phosphorylation levels of p38 and Erk1/2 were analysed at 30 minutes after TGF-β1 stimulation. The time point of 30 minutes was determined by a time course experiment (Supplementary Figure 2A). As expected, our data showed that IDH1 knockdown increased the phosphorylation levels of p38 and Erk1/2 induced by TGF-β1; in contrast, IDH1 overexpression decreased the phosphorylation levels of p38 and Erk1/2 (Figure 3C).

To determine how IDH1 affects TGFBR, the mRNA levels of TGFBR1 and TGFBRII were first analyzed by realtime-PCR. The data did not show that IDH1 affected their mRNA levels (Figure 3D). To determine whether IDH1 regulates the expression of TGFBR1 or TGFBRII, the protein levels of TGFBR1 and TGFBRII were analyzed by Western blot. Our data showed that IDH1 knockdown did increase the protein levels of TGFBR1 and TGFBRII while IDH1 overexpression decreased their protein levels (Figure 3E). To confirm the regulation of IDH1on TGFBR1 and TGFBRII, the protein levels of TGFBR II and phosphorylated TGFBR1 were examined by immunofluorescence. The microscopy pictures showed that TGF-β1 activates TGFBR1, which is indicated by the foci of p-TGFBR 1; and the downregulation of IDH1 increased the foci number of p-TGFBR 1. Moreover, IDH1 downregulation also increased the the foci number of TGFBRII (Figure 3F). In contrast, IDH1 overexpression decreased the protein levels of p-TGFBR 1 and TGFBRII, which was reflected by the foci number (Supplementary Figure 2B). These observations suggest that IDH1 regulates the protein levels of TGFBR1 and TGFBRII, not their mRNA levels.

To further confirm whether IDH1 regulates TGFBR degradation, the protein levels of TGFBR1 and TGFBR II were analyzed in fibroblasts overexpressing or depleted of IDH1. All these cells were treated with protein synthesis inhibitor cycloheximide before analysis. Western blots showed that the time for TGFBR1 degradation was prolonged to 6 hours in fibroblasts with IDH1 knockdown, longer than that in control fibroblasts; and the TGFBRII protein degraded slower in IDH1 knockdown fibroblasts. In contrast, the TGFBR1 and TGFBR II proteins degraded faster in fibroblasts overexpressing IDH1 (Figure 3G). These results suggested that downregulation of IDH1 suppressed the degradation of TGFBR.

### Downregulation of IDH1 reduces Cav1 expression through α-KG-mediated epigenetic regulation

**Figure 4:**
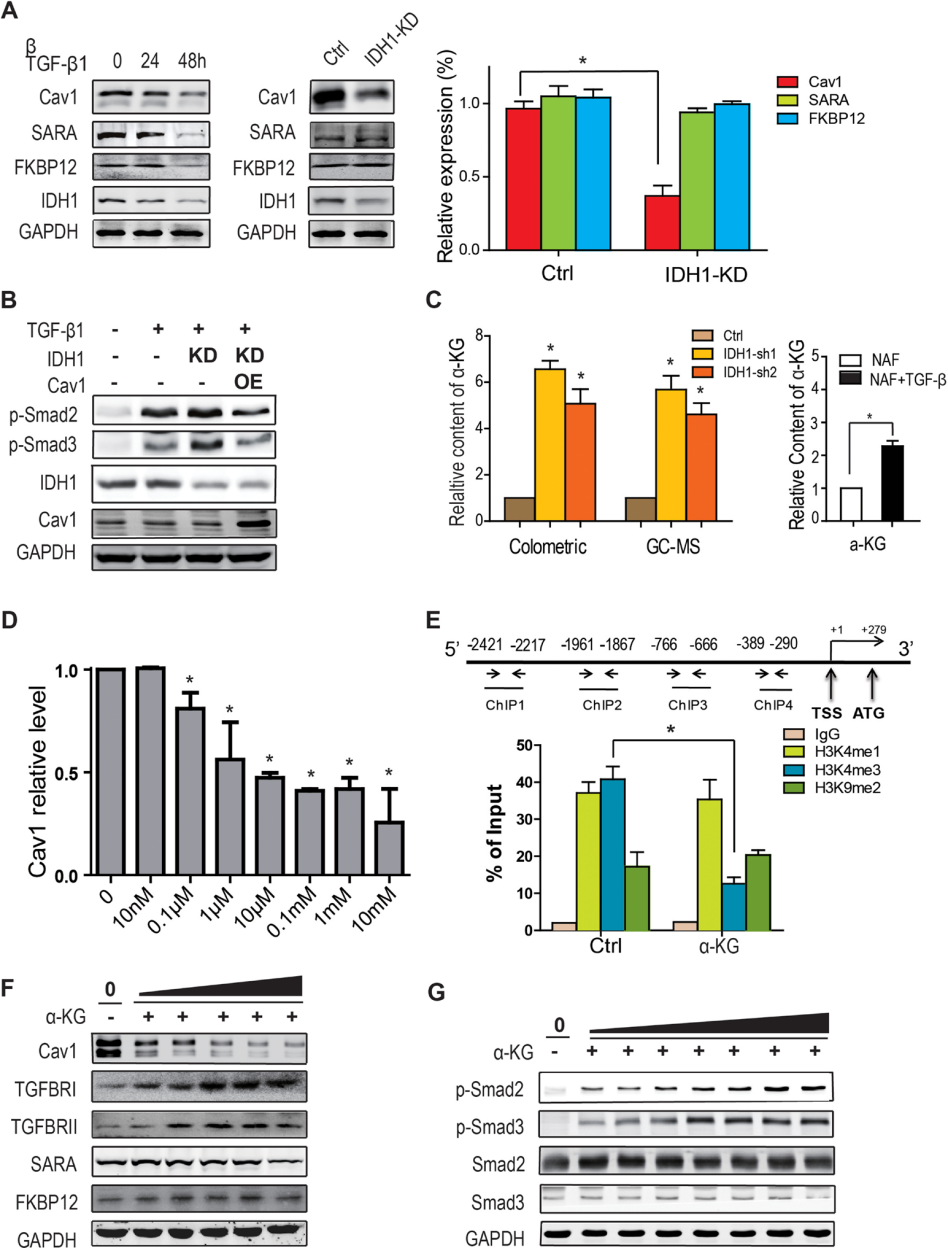
Downregulation of IDH1 reduces Cav1 expression through α-KG-mediated epigenetic regulation (**A**) Western blots showing the downregulated protein levels of Cav1 in IDH1-knockdown fibroblasts. The protein levels and mRNA levels of Cav1, FKBP12 and SARA were detected in primary fibroblasts treated with TGF-β1 (8 ng/ml) for 24 or 48 hours (*n* = 3, *p < 0.01). (**B**) Overexpression of Cav1 significantly attenuated the TGF-β1-induced phosphorylation of Smad2/3 in 293T cells depleted of IDH1. The plasmids containing IDH1 shRNA1 or/and Cav1 coding sequences were transfected into 293T cells, which were treated with TGF-β1 for 1 hour before harvest. (**C**) α-KG was increased in IDH1-knockdown fibroblasts and TGF-β-treated fibroblasts. The concentrations of α-KG in primary or IDH1-knockdown fibroblasts were analysed by GC-MS and α-KG assay kit (*n* = 3, *p < 0.01). (**D**) α-KG decreased the expression of Cav1. Primary fibroblasts were treated with α-KG at the indicated doses (10 nM, 0.1 μM, 1 μM, 10 μM, 0.1 mM, 1 mM, and 10 mM) for 24 hrs. The mRNA levels were normalised to GAPDH (*n* = 3, *p < 0.01). (**E**) ChIP assay showing that the trimethylation level of H3K4 decreased in the *Cav1* promoter region after α-KG treatment. Chromatin IP assays of HEK293T cells, with or without 1 mM α-KG treatment for 24 hours, were performed using antibody against H3K4me1, H3K4me3, H3K9me2 or IgG and analysed by real-time PCR (*n* = 3, *p < 0.01). (**F**) The protein level of Cav1 gradually decreased with increasing α-KG concentration. The primary fibroblasts were treated for 48 hours with the indicated doses of α-KG (left to right: 1 μM, 10 μM, 0.1 mM, 1 mM, and 10 mM). (**G**) The phosphorylation of Smad2 and Smad3 was also gradually increased with increasing α-KG concentration. Primary fibroblasts were treated for 1 hour with α-KG at the indicated doses (left to right: 10 nM, 100nM, 1 μM, 10 μM, 0.1 mM, 1 mM, and 10 mM).

The above data demonstrates that IDH1 regulates TGFBR degradation; however, the mechanism by which IDH1 regulates TGFBR degradation is not clear. Firstly, several proteins affecting TGFBR, such as Smad anchor for receptor activation (SARA), FK506 binding protein 1A (FKBP12) and caveolin 1 (Cav1), were examined in TGF-β1-treated fibroblasts. The data from Western blot analysis showed that the expression of all three proteins decreased in TGF-β1-treated fibroblasts, but only the Cav1 protein level was reduced in fibroblasts depleted of IDH1 (Figure 4A). Moreover, the quantitative PCR result confirmed this finding (Figure 4A). These observations suggested that downregulation of IDH1 suppressed the expression of Cav1, but not of SARA or FKBP12.

To further determine whether Cav1 mediates IDH1-enhanced Smad signalling, Cav1 was overexpressed in 293T cells depleted of IDH1. After TGF-β1 stimulation, Smad2/3 were phosphorylated, and this phosphorylation was increased in 293T cells depleted of IDH1. However, overexpression of Cav1 significantly attenuated the phosphorylation of Smad2/3 after TGF-β1 stimulation, especially when compared to the cells depleted of IDH1 (Figure 4B). These observations suggested that Cav1 mediated the regulation of IDH1 on TGFBR.

IDH1 is a critical metabolic enzyme that converts isocitrate into α-ketoglutarate (α-KG), which is an allosteric regulator of a variety of dioxygenases, many of which are regulators of gene transcription, i.e., histone demethylases. To test this hypothesis, the concentration of α-KG was analysed in control fibroblasts or fibroblasts depleted of IDH1 using GC-MS or α-KG assay kit. Our data showed that the concentration of α-KG did increase in IDH1-knockdown fibroblasts, which was further supported by the data that TGF- β increased cellular α-KG content (Figure 4C). This increase could be due to an enhanced glutamine metabolic pathway [15]. Moreover, the expression of Cav1 in primary fibroblasts treated with dimethyl-α-KG at the indicated concentration was analysed by quantitative PCR. Our data showed that 0.1 μM of α-KG was enough to suppress Cav1 expression, and Cav1 expression reached its lowest peak at a concentration of 10 μM α-KG (Figure 4D), which suggested that IDH1 regulated Cav1 expression through α-KG.

We proposed that increased α-KG activated histone demethylase, which might decrease the tri-methylation level of histone H3K4 and increase the di-methylation of H3K9. In turn, decrease of H3K4 tri-methylation and increase of H3K9 di-methylation would suppress gene expression at its locus [16]. To determine whether α-KG regulates the methylation level of histone H3K4 and h3K9 in the promoter region of *Cav1*, chromatin immunoprecipitation (ChIP) assays using a specific antibody against H3K4me1, H3K4me3 or H3K9me2 were performed. The results from the ChIP assays showed that α-KG did decrease the trimethylation level of H3K4 (*P* < 0.01) but not the dimethylation of H3K9 (*P* > 0.05) in the locus of the *Cav1* promoter (Figure 4E). These data suggested that downregulation of IDH1 reduced Cav1 expression through α-KG, at least partially.

To further confirm that α-KG regulates TGFBR degradation through Cav1, the protein levels of Cav1 and TGFBR1/TGFBRII were analysed in parallel in fibroblasts treated with the indicated doses of α-KG. Our data showed that the protein level of Cav1 gradually decreased with increasing α-KG concentration and was significantly reduced at 0.1 mM of α-KG. In contrast, the protein level of TGFBR1 and TGFBRII gradually increased with increasing α-KG concentration and was significantly ascended at 0.1 mM of α-KG (Figure 4F). In time-course experiments, Cav1 expression gradually decreased in fibroblasts treated with 1 mM α-KG while TGFBR1 expression gradually ascended (Supplementary Figure 3A). Meanwhile, the phosphorylation of Smad 2 and 3 also gradually went up with increasing α-KG concentration one hour after α-KG treatment (Figure 4G); the time point of one-hour was determined by a time-course experiment (Supplementary Figure 3B). In brief, these observations suggested that downregulation of IDH1 feedback increased the cellular concentration of α-KG; the latter, in turn, suppressed TGFBR degradation through Cav1 and enhanced TGF-β signalling.

### Downregulation of IDH1 enhanced TGF-β signalling

**Figure 5:**
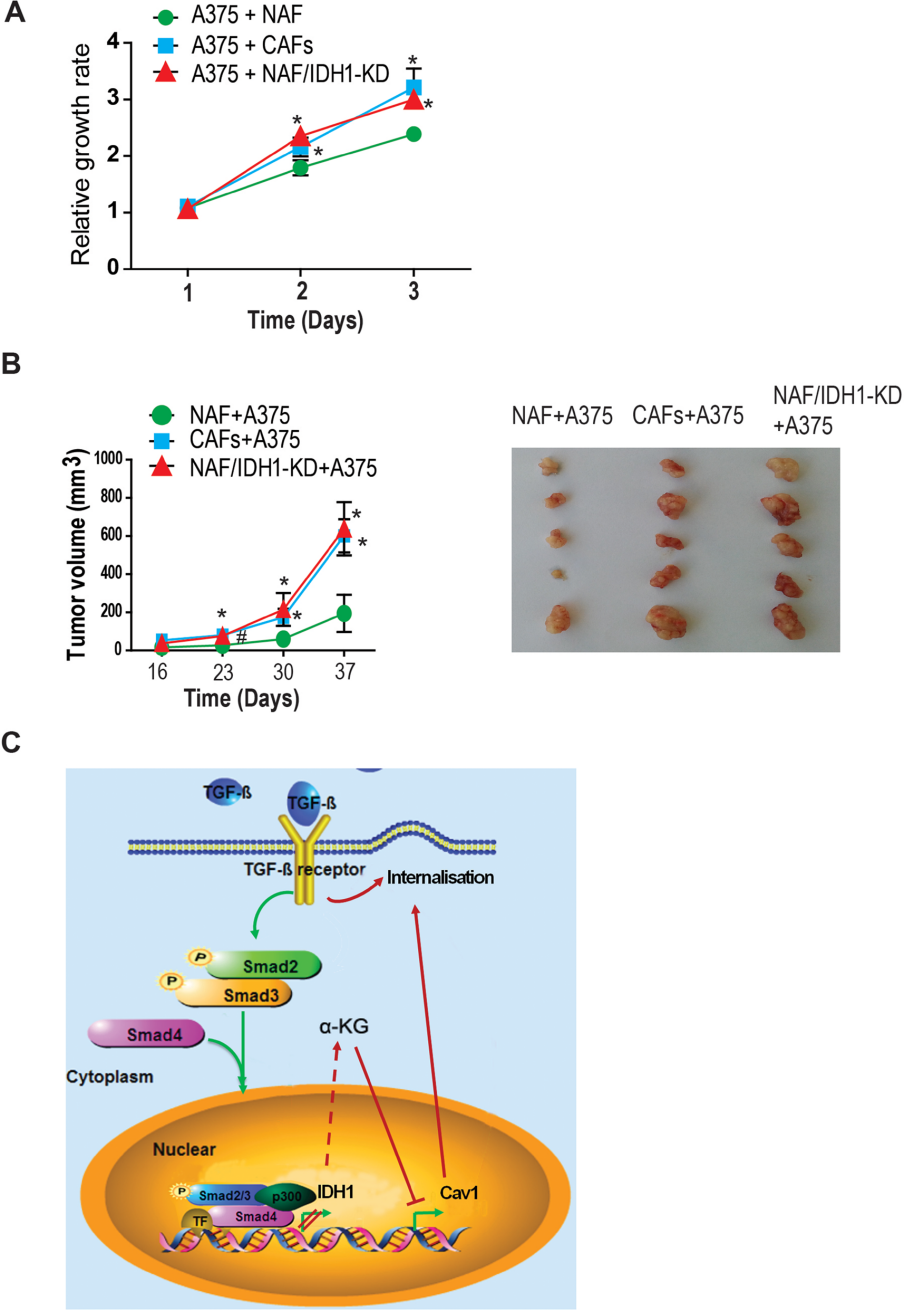
Downregulation of IDH1 enhanced TGF-β-induced fibroblast activation and epithelial-mesenchymal transition (**A**) The tumour promoting effect of fibroblasts knockdown IDH1 were analysed in *in vitro* or *in vivo*. A375 cells were co-cultured with the media from different fibroblasts, and the growth rate of A375 was measured using CCK8 assay at the indicated time points (1, 2, 3 days) (*n* = 3, *p < 0.01). (**B**) Tumor xenografts were generated by subcutaneously co-injecting A375 cells with human NAFs or CAFs. The graphic shows the tumor growth curve (^#^*p* < 0.05, *p < 0.01). The pictures are representative tumours from three groups. (**C**) The working model about the IDH1-Cav1 feedback regulation of Smad signalling.

To determine the biological significance of IDH1-Cav1 feedback regulation, the tumour promoting effects were analyzed in IDH1-knockdown fibroblasts. The proliferation rate of A375 cells co-cultured with the media from fibroblasts depleted of IDH1 was firstly measured. Our data showed that A375 cells co-cultured with IDH1-knockdown fibroblasts grew faster than co-cultured with control fibroblasts. The proliferation rate of A375 cells co-cultured with IDH1-knockdown fibroblasts was similar as co-cultured with cancer-associated fibroblasts (CAF) (Figure 5A). Moreover, the *in vivo* tumour promoting effect of fibroblasts depleted of IDH1 was observed in xenografted tumour mice. The tumours in IDH1 knockdown group grew faster than control group (*P* < 0.05), the median of tumour size were no significant difference between IDH1-knockdown group and CAFs group (*P* > 0.05) (Figure 5B).

In brief, our study demonstrated that TGF-β signalling downregulated IDH1 expression through canonical Smad pathway; and downregulation of IDH1 suppressed Cav1 expression through α-KG-dependent epigenetic regulation; eventually, the decrease of Cav1 expression interrupted TGFBR degradation and enhanced Smad signalling (Figure 5C).

## DISCUSSION

It is well known that tumour growth is determined not only by malignant cancer cells themselves but also by the tumour stroma. Activated fibroblasts, also called cancer-associated fibroblasts (CAFs), are the main components of the tumour stroma. Increasing data have demonstrated that CAFs play important roles in tumour growth and progression. Tumour peripheral resting fibroblasts is believed to be a major origin of CAFs, and tumour-secreted cytokines, such as TGF-β, are important revulsants for fibroblasts activation [17–20].

Once the receptor is activated, TGF-β signalling is mediated through canonical and non-canonical pathways to regulate transcription, translation, microRNA biogenesis, protein synthesis, and post-translational modifications [21–23], and eventually changes cell functions, such as fibroblasts activation and tumour cells EMT transition.,. Our data showed that the expression of IDH1 was downregulated by TGF-β signalling, which is consistent with the recent observations that TGF-β signalling participates in metabolism regulation. However, the regulation of IDH1 was not likely associated with CAFs formation since PDGF treatment didn't change the IDH1 expression. We will further investigate the detail mechanism underlying TGF-β-induced IDH1 downregulation.

TGFBR activity is finely tuned by SARA, FKBP12 and Cav1. SARA directly interacts with Smad2/3, recruits Smad2 to the TGFB receptor, and plays an essential role in TGF-beta-induced Smad2 activation. SARA also functions as an anchor for the catalytic subunit of protein phosphatase 1 (PP1c) and is involved in the dephosphorylation of TGFBR I [25]. FKBP12, a 12-kDa FK506-binding protein, directly interacts with the type I TGF-β receptor and acts as an inhibitor of TGF-beta signalling. FKBP12 binds to the glycine- and serine-rich motif (GS motif) of the type I TGFBR with Smad7, another inhibitory molecule of TGFBR. Upon stimulation with ligand, FKBP12 is released from the receptor to fully propagate the signal [26]. In addition, TGFBR is located in cellular caveolae, where it functionally interacts with Cav-1 through its scaffolding domain. Through caveolin-1 lipid rafts, TGFBR undergoes rapid degradation, which effectively decreases TGF-β signalling [26]. In this study, we demonstrated that IDH1 knockdown suppressed the expression of Cav1 through α-KG-mediated epigenetic regulation while TGF-signalling was enhanced when the protein level of Cav1 was reduced, which was consistent with the data from other groups.

Alpha-KG is a substrate of isocitrate dehydrogenase or glutamic pyruvate transaminase, it is believed that the glutamate pathway is complementally activated when the conversion of isocitrate into α-KG is decreased due to the inhibition of expression level or kinase activity of isocitrate dehydrogenase. IDH1 downregulation-triggered increase of α-KG concentration in fibroblasts is possibly due to the similar mechanism. In general, increased α-KG promotes the activity of a range of dioxygenases, including lysine-specific demethylases, which are KDM family members. Our data showed that the trimethylation of histone H3K4 was decreased in the locus of *Cav1* promoter in IDH1 knockdown cells and resulted in the suppression of Cav1 expression. These observations suggest that α-KG promotes the activity of KDMs, which, in turn, declines the trimethylation of histone H3K4. The specific member of KDM family that is responsible for the demethylation of trimethylated H3K4 in the promoter region of *Cav1* will be determined in our future study.

In brief, our data demonstrated that the metabolic enzyme IDH1 regulated TGF-β signalling through α-KG, and the TGFBR-IDH1-Cav1 feedback loop enhanced TGF-β signalling, which illustrated a novel regulation network between cell signalling and cellular metabolism.

## MATERIALS AND METHODS

### Cell culture and plasmid construction

The HEK293T cell line was obtained from ATCC (Manassas, VA, USA) and cultured in DMEM medium with 10% FBS and 100 μg/ml penicillin-streptomycin. Human normal primary fibroblasts were isolated from foreskin and cultured in DMEM with 15% FBS and 100 μg/ml penicillin-streptomycin. To construct the pHRSIN-IDH1 plasmid, a fragment of the IDH1 mRNA (GenBank accession number: NM_005896.3) was amplified and cloned into the lentivirus vector pHRSIN. The shRNA plasmids of IDH1 were gifts from Shimin Zhao (Fudan University, Shanghai, China). The shRNA plasmids of Smad2, Smad3 and Smad4 were constructed previously [20].

### Isolation of human normal primary fibroblasts

This study was approved by the Ethical Review Board of the Medical Faculty of the Shanghai Jiao-Tong University School of Medicine. Human normal primary fibroblasts were collected after written consent from children aged 7 to 12 years at the affiliated Xinhua Children's Hospital of the Shanghai Jiao Tong University School of Medicine. After posthectomy, the foreskins were immediately transported to the laboratory in sterile PBS buffer containing 1% penicillin/streptomycin on ice. After 2 washes, the foreskins were minced using sterile scalpels and scissors and then digested with 0.1% type I collagenase and trypsin in a shaking water bath at 37 °C for 30 min. After digestion, the tissue was filtered with a 400-mesh sieve, and the filtrate was centrifuged at 1000 × *g* for 10 min. Cells obtained from the pellet were cultured with DMEM containing 10% FBS for 2 h; the attached cells, verified by F-actin staining, were fibroblasts. After 3 passages, the cells were frozen in liquid nitrogen for further experiments.

### Lentiviral transduction and generation of stable cell lines

The IDH1 ectopic-expression plasmid and shRNA-targeting plasmids were transfected into 293T cells with pSPAX2 and pMD2G to generate the respective lentiviruses. To obtain stable cell lines, cells at low confluence (20%~30%) were infected overnight with lentiviral supernatants diluted 3:1 in normal culture media in the presence of 10 ng/ml polybrene (Sigma). Human fibroblasts were subjected to puromycin selection for 1 week after transfection and then propagated before use. Puromycin at 500 ng/ml was used to maintain the stable cells.

### Western blotting and nuclear/cytosolic extracts

The cells were immediately placed on ice and washed with ice-cold PBS. Total protein extract was prepared with the appropriate amount of RIPA lysis buffer (25 mM Tris-HCl at pH 7.5, 2 mM EDTA, 25 mM NaF and 1% Triton X-100) containing 1 X protease inhibitor mixture (Roche, Basal, CH, Switzerland) and 1 X PMSF. The nuclear/cytosolic extraction method was described previously [27]. The proteins were resolved on 7-15% SDS-polyacrylamide gels and transferred by electroblotting to nitrocellulose membranes (Bio-Rad, Hercules, CA, USA). The membranes were blocked with 5% non-fat dry milk in TBST for 1 hour. Proteins of interest were detected with specific antibodies, blots were scanned using an Odyssey infrared imaging system (LI-COR), and proteins were quantitatively analysed using the Odyssey software.

### Reagents and antibodies

Human recombinant TGF-β1, PDGF and BMP4 were purchased from Biolegend (San Diego, CA). Fast-start universal SYBR Green master mix was from Roche, and sb431542, MG132 and chloroquine were from Sigma. The antibodies used for immunoblotting (IB): GAPDH 1:10000 (Sigma), β-actin 1:5000 (Santa Cruz),Smad2 1:1000 (Cell Signalling), Smad3 1:1000 (Cell Signalling), p-Smad2 (Ser465/467) 1:1000 (Cell Signalling), p-Smad3 (Ser423/425) 1:500 (Cell Signalling), IDH1 1:1000 (Origene), SP1 1:1000 (Sigma), α-tubulin 1:1000 (Santa Cruz), p-p38 1:1000 (Cell Signalling), p38 1:1000 (Cell Signalling), p-Erk1/2 1:2000 (Cell Signalling), Erk1/2 1:1000 (Cell Signalling), S100A4/FSP1 1:500 (Abnova), TGFBRI 1:1000 (Cell Signalling), TGFBRII 1:1000 (Abcam), p-TGFBRI 1:200 (Abcam), Cav1 1:1000 (ProteinTech), FKBP12 1:1000 (ProteinTech), and SARA 1:1000 (ProteinTech).

### RNA extraction and real-time PCR

The culture medium was removed, and the cells were immediately washed with ice-cold PBS. Subsequently, 1 ml of TRIzol reagent was added, and total cellular RNA was extracted using the acid guanidinium thiocyanate-phenol-chloroform method. Total RNA (1 μg) was used as a template for an MMLV-RT reverse transcriptase reaction, which was performed according to the manufacturer's instructions. Real-time quantitative reactions were set up in triplicate in a 96-well plate, and each reaction contained 1 μl of cDNA and the SYBR Green PCR mix, to which gene-specific forward and reverse PCR primers were added. Melting curves were analysed to verify the specificity of the RT-PCR reaction and the absence of primer dimer formation. The following primers were used: GAPDH sense: 5- ACCCAGAAGACTGTGGATGG -3, antisense: 5- CAGTGAGCTTCCCGTTCAG -3; IDH1 sense: 5-CACTACCGCATGTACCAGAAAGG -3, antisense: 5-TCTGGTCCAGGCAAAAATGG -3; TGFBRI sense: 5- GCTGACATCTATGCAATGGG -3, antisense: 5- TTTCTTCAACCGATGGATCA -3; TGFBRII sense: 5- CCGCTGCATATCGTCCTGTG -3, antisense: 5- AGTGGATGGATGGTCCTATTAC -3; Cav1 sense: 5- GGCAGTTGTACCATGCATTA -3, antisense: 5- ATTTTCCCAACAGCTTCAAA -3; SARA sense: 5- TGTGTTGGATTGGCAGATG -3, antisense: 5- GAAACACCTGGGTCTTGCAT -3; FKBP12 sense: 5- GGGATGCTTGAAGATGGAAA -3, antisense: 5- CACATCGAAGACGAGAGTGG -3.. The mRNA levels of the target genes were normalised to GAPDH. Each target was measured in triplicate, and data were analysed using GraphPad Prism 5.

### Immunofluorescence staining

Immunofluorescence staining was performed on cultured cells. After fixation and permeabilization, the cells were incubated with primary antibodies at 4°C overnight, followed by the appropriate Alexa Fluor 488/594-conjuated secondary antibodies. The cells were visualized by fluorescence microscopy. The mean fluorescence index was the average intensity analyzed in 100 cells using Image J (*p < 0.01, *n* = 100).

### ChIP assays

HEK293T cells with or without α-KG treatment were fixed in 1% formaldehyde for 20 minutes at 37 °C and quenched in 0.125 M glycine. Chromatin was immunoprecipitated from sonicated cell lysates using the H3K4me1, H3K4me3, H3K9me2 antibody or IgG and quantified using SYBR Green Real-time PCR analysis. The fold enrichment was calculated based on the Ct using the equation 2^-Δ(ΔCt)^, where ΔCt = ΔCtIP - ΔCtInput and Δ(ΔCt) = ΔCtantibody - ΔctIgG. The primer sequences were as follows: Cav1-ChIP1-F: 5′- CTTGAGGCCAGGAGTTTGAGAC-3′, Cav1-ChIP1-R: 5′- GCACCACCACACCCTGCTAA-3′; Cav1-ChIP2-F: 5′- CGAGATTGCTTTCCCTCGGT-3′, Cav1-ChIP2-R: 5′- GGAACACAGAGGGAGCTTGTC-3′; Cav1-ChIP3-F: 5′- AGCCCCAGATTCAGGAACAGAC-3′, Cav1-ChIP3-R: 5′- TGTGCTTGGCTGTGAGGAAA-3′; and Cav1-ChIP4-F: 5′- AGTACACCACAGGCACCCAC-3′, Cav1-ChIP4-R: 5′- GGGAGGGATGAAAGACGGCT-3′.

### GC-MS and Colorimetric Assay for α-KG Quantification

The intracellular content of α-KG was analyzed by GC-MS as previously described (Chan et al., 2009). The cells (1 × 10^8^) were harvested and suspended in chloroform-methanol-water (2:1:1, v/v/v). The derivatized sample was injected into a Shimadzu QP 2010 GC tandem quadrupole mass spectrometer. The GC separation was performed on an Agilent DB-5 mass spectrometer fused silica capillary column (30 m × 0.25 mm × 0.25 μm). The column temperature was 70°C for the first 3 min and then increased at 5°C/min to 310°C for 5 min. The injection temperature was set as 300°C, and the injection volume was 1 μl with a 10:1 split ratio. Helium (99.9995%) was applied as a carrier gas. The column flow was 1.2 ml/min, and the column was equipped with a linear velocity control. The mass spectra scanning scope was set to 33−600 m/z in the full-scan mode with a scan speed of five scans s^−1^ and a solvent cut time of 5.6 min based on the retention time of the pyridine solvent. The temperatures of the interface and the ion source were adjusted to 280°C and 240°C, respectively. The detector voltage was maintained at 1.2 kV, and the electron impact (EI) model was selected to achieve ionization of the metabolites at 70 eV. α-KG assay was performed according to the instructions of the α-KG Assay Kit (BioVision, catalog #K677-110).

### Tumor xenografts

Six-week-old BALB/c nude mice were purchased from Shanghai Laboratory Animal Center. The animals were operated according to the protocol approved by the Institutional Animal Care and Use Committee of the Shanghai Jiao Tong University School of Medicine. Tumor xenografts were generated by subcutaneously co-injecting A375 cells with human non-activated fibroblasts (NAFs) or CAFs (cancer-associated fibroblasts) into the armpits bilaterally, the ratio of A375 to fibroblasts was 1:3, and the total cell number in each injection was 4 × 10^6^. NAFs were depleted of IDH1 or infected by empty viruses. The tumor volumes in the three groups were determined weekly using digital caliper measurements and the following formula: tumor volume (mm^3^) = ½ × longest diameter × shortest diameter^2^. After 9 weeks, the mice were sacrificed and the tumors were excised.

### Statistical analysis

The animal data are presented as the medians ± SD, whereas other data are presented as the means ± SD. All data are representative of at least three independent experiments. The differences between groups were assessed by Student's *T*-test; all reported differences are *p* < 0.01 unless otherwise stated.

## SUPPLEMENTARY MATERIALS FIGURES


