# Supplementary material for: TGFBR-IDH1-Cav1 axis promotes TGF-β signalling in cancer-associated fibroblast
Source: Oncotarget. 2017 Sep 13;8(48):83962–74. doi: 10.18632/oncotarget.20861 (PMC5663568; doi:10.18632/oncotarget.20861)
Supplement: Supplementary file 1 [file oncotarget-08-83962-s001.pdf]

TGFBR-IDH1-Cav1 axis promotes TGF-β signalling in cancer-associated fibroblast

SUPPLEMENTARY MATERIALS

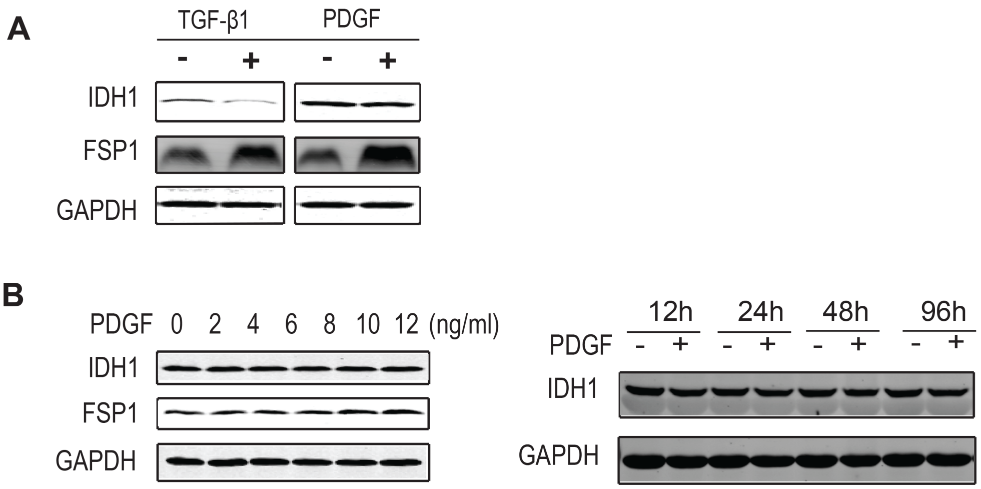

Supplementary Figure 1: The protein level of IDH1 is downregulated in fibroblasts treated by TGF-β1, but not PDGF. (**A**) The primary fibroblasts were treated with 8 ng/ml TGF-β1 or 10 ng/ml PDGF twice for 48 hours. FSP1 is a marker of CAFs. (**B**) Time course and dose response experiment were performed on fibroblasts treated with PDGF. The left Western blot show the protein level of IDH1 in fibroblasts treated with the indicated dose of PDGF (0, 2, 4, 6, 8, 10, 12 ng/ml) for 24 hours. The right Western blot show the protien level of IDH1 at the indicated time points (12, 24, 48, 96 hours) after 10 ng/ml PDGF treatment.

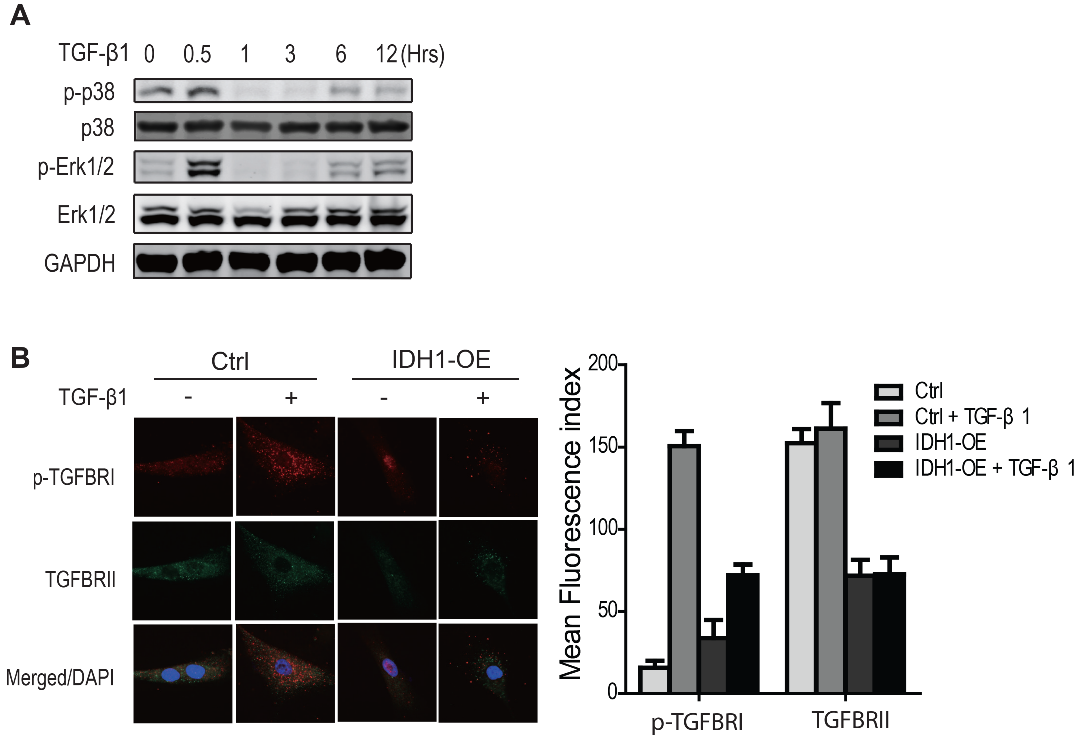

Supplementary Figure 2: (**A**) The dynamic changes of non-canonical TGF-β signaling upon TGF-β1 stimulation A. time-course experiment was performed to analyse the dynamic changes of the phosphorylation levels of p38 and Erk1/2 at the indicated times (0, 0.5, 1, 3, 6, and 12 hrs) after TGF-β1 stimulation. (**B**) The immunofluorescence staining of phospho-TGFBR1 and TGFBRII. Fibroblasts were fixed, but did not permeabilized; pictures are representative confocal microscopy images (X 40). The mean fluorescence index was the average intensity analyzed in 100 cells using Image J (*$p < 0.01$, $n = 100$).

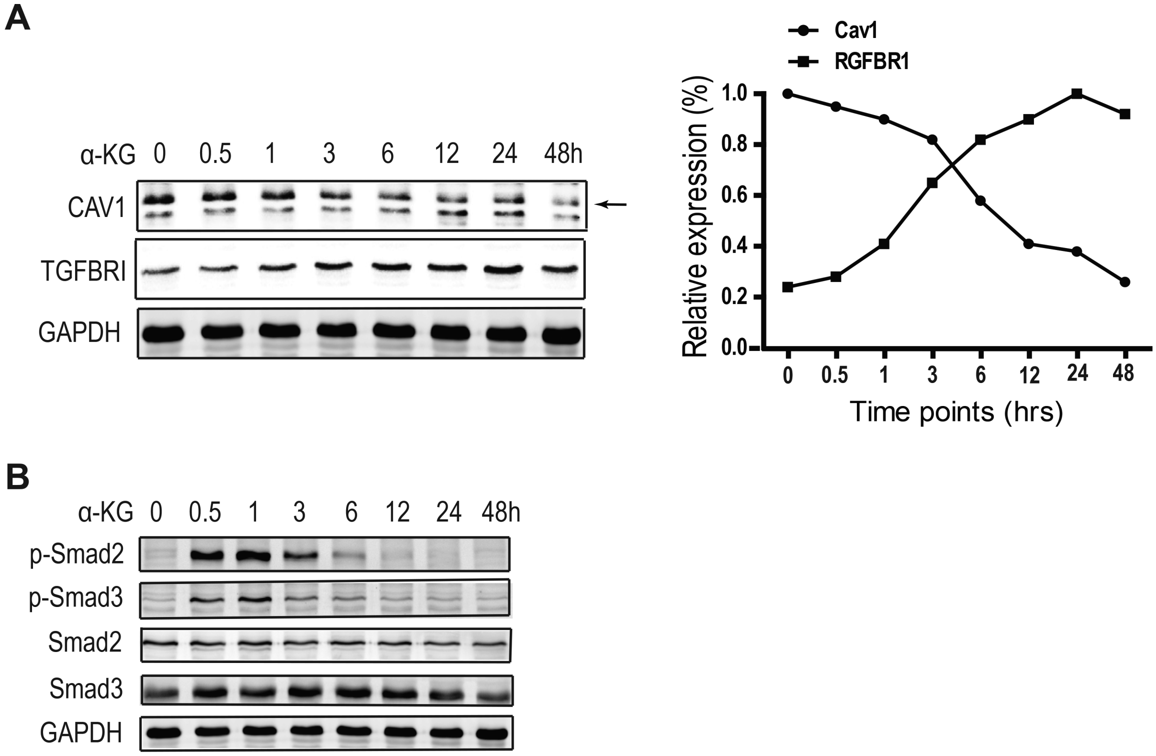

Supplementary Figure 3: α-KG suppresses Cav1 expression and inhibits the internalization of TGFBR1. (**A**) the protein level of Cav1 and TGFBR1 were detected in primary fibroblasts treated with 1 mM α-KG at the indicated time points (0, 0.5, 1, 3, 6, 12, 24, 48 hours). (**B**) the phospho- and total protien level of Smad2 and Smad3 were analyzed in fibroblasts treated with 1 mM (α-KG) at the indicated time points (0, 0.5, 1, 3, 6, 12, 24, 48 hours).
